# Experimental and Computational Approaches for Non-CpG Methylation Analysis

**DOI:** 10.3390/epigenomes6030024

**Published:** 2022-08-16

**Authors:** Deepa Ramasamy, Arunagiri Kuha Deva Magendhra Rao, Thangarajan Rajkumar, Samson Mani

**Affiliations:** Department of Molecular Oncology, Cancer Institute (WIA), 38, Sardar Patel Road, Chennai 600036, India

**Keywords:** non-CpG methylation, techniques, methods, computational approaches, mCpH

## Abstract

Cytosine methylation adjacent to adenine, thymine, and cytosine residues but not guanine of the DNA is distinctively known as non-CpG methylation. This CA/CT/CC methylation accounts for 15% of the total cytosine methylation and varies among different cell and tissue types. The abundance of CpG methylation has largely concealed the role of non-CpG methylation. Limitations in the early detection methods could not distinguish CpG methylation from non-CpG methylation. Recent advancements in enrichment strategies and high throughput sequencing technologies have enabled the detection of non-CpG methylation. This review discusses the advanced experimental and computational approaches to detect and describe the genomic distribution and function of non-CpG methylation. We present different approaches such as enzyme-based and antibody-based enrichment, which, when coupled, can also improve the sensitivity and specificity of non-CpG detection. We also describe the current bioinformatics pipelines and their specific application in computing and visualizing the imbalance of CpG and non-CpG methylation. Enrichment modes and the computational suites need to be further developed to ease the challenges of understanding the functional role of non-CpG methylation.

## 1. Introduction

DNA methylation is the well-known epigenetic modification that occurs at the C5 position of cytosine [1]. About 85% of cytosine DNA methylation (5-methylcytosine or 5-mC) occurs at CG dinucleotides, functionally known to control gene expression [2]. Conversely, 15% of methylation which occurs in the cytosine of CC, CT, and CA is grouped as non-CpG methylation or mCpH (H = A, T, C) [3,4,5]. Most of the cell types carry CpG methylation, but the non-CpG methylation is abundant in neurons and oocytes, moderately distributed in embryonic stem cells, and almost negligible in other cell types [6,7]. During DNA replication, DNMT1 maintains CpG methylation where the non-CpG methylation is eventually lost during development and cell division [8,9]. However, non-CpG methylation can be imparted by de novo methylases such as DNMT3a, DNMT3b, and DNMT3L complexes that are mostly observed in differentiating cells. Non-CpG methylation equally contributes to gene regulation; hence, its imbalance can lead to disease pathogenesis. Several human diseases such as Rett syndrome, Alzheimer’s, Parkinson’s, and other lifestyle diseases such as cancer have been associated with altered levels of non-CpG methylation [10,11,12,13,14].

Initial studies reported non-CpG methylation as a novel modification in the early 1980s [15]. However, the potential functional role of non-CpG methylation in gene regulation was revealed only after the 2000s. Ramsahoye et al., (2000) were the first to describe non-CpG methylation as an independent epigenetic modification and its significance in developmental biology. The study provided evidence for the regulation of de novo methylase Dnmt3a in imparting non-CpG methylation marks in embryonic stem cells (ESCs) [16]. Later in 2001, Malone et al. described the association of mCpH with B-cell lymphoma [17]. Despite finding the non-CpG methylation content in several cells and tissue types, the genomic distribution and its precise contribution to gene regulation are not completely understood. Earlier methods were designed for detecting CpG methylation which either discounted non-CpG methylation or over-represented CpG methylation alone. However, these methods do not contribute to the genome-wide distribution of non-CpG methylation. Advancements in enrichment strategies and sequencing techniques aided in the detection of non-CpG methylation at loci-specific resolution. Yet, developing a simple and sensitive method that can exclusively deduce non-CpG methylation can help in understanding the precise role in development and disease biology.

In this review, we extensively focus on the existing experimental and computational approaches for the detection of non-CpG methylation. This review discusses majorly the development of experimental methods including enzyme-based enrichment methods; antibody-based strategies. followed by high throughput sequencing in profiling the genome-wide non-CpG methylation in several tissue types. Moreover, the advancement of single-molecule real-time sequencing in the detection of non-CpG methylation was also discussed. The advantages of computational approaches in differentiating non-CpG methylation were explained. Additionally, we also focused on the drawbacks and limitations in distinguishing mCpH from mCpG in the human genome. Therefore, unraveling the challenges in the technical strategies would strengthen us with the promising future for detecting non-CpG methylation in human disease discovery and therapeutic approaches.

## 2. Experimental Approaches for Non-CpG Methylation Analysis

### 2.1. Conventional Methods

The detection of non-CpG methylation happened incidentally during the enrichment of CpG methylation. Initial studies used the nick labeling technique to profile non-CpG methylation which reported the information on nucleotide sequences in dinucleotide composition [15]. Since the short length of the nucleotides was a major drawback of the technique, a dual label nearest neighbor assay was developed which involved the labeling of DNA with two different isotopes, followed by the treatment with restriction enzymes. The dual label nearest neighbor assays (NNA) included [32P] labeled dATPs, dTTPs, and dCTPs that were incorporated to detect non-CpG methylation and [33P] labeled dGTPs to detect CpG methylation. After “fill-in”, the labelled genomic DNA was digested with the methylation-specific restriction enzymes followed by HPLC for quantification. Thus, NNA could type both CpG and non-CpG methylation [16,18,19]. However, the major limitations of NNA were the background radioactivity and a higher amount of genomic DNA as starting material. Despite these limitations, this is the first technique without the involvement of sequencing strategies that quantified the non-CpG methylation levels.

### 2.2. Enzyme-Based Enrichment Methods

**Figure 1:**
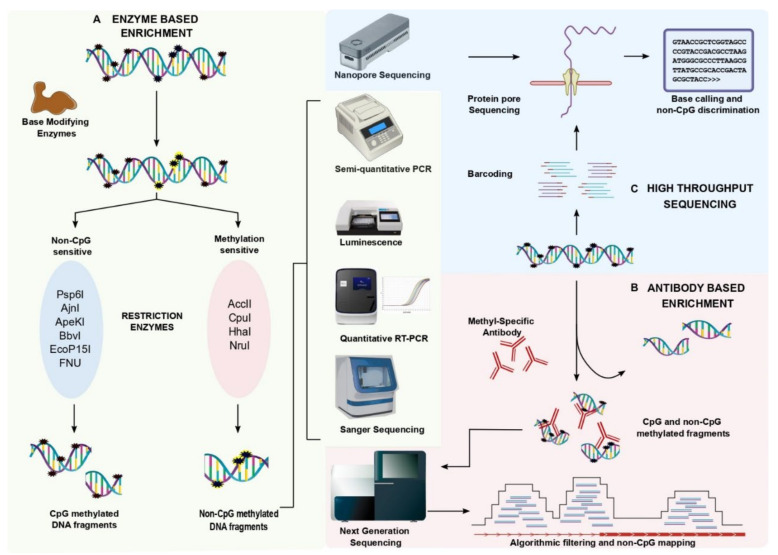
Enrichment strategies that are involved in non-CpG methylation detection and analysis. (**A**) Enzyme-based enrichment techniques: Enzyme-based enrichment approaches are classified based on the methylation-sensitive and -insensitive restriction enzymes. The targeted endonuclease activity eliminates CpG methylation and enriches non-CpG methylation that can be deduced by other methods such as semi-quantitative PCR, luminescence, quantitative PCR, and also read through Sanger sequencing. (**B**) Antibody-based enrichment techniques: Antibody-based enrichment strategies exploit the use of 5-methylcytidine antibodies that are specific to 5-mC to capture the methylated cytosines irrespective of their adjacent moiety. The captured methylated cytosines have both CpG and non-CpG methylation. Algorithmic filtering should be employed to the reads that are obtained from sequencing to differentiate non-CpG methylation from CpG methylation. Also, methods such as semi-quantitative PCR, luminescence, quantitative PCR, and Sanger sequencing can be used to detect non-CpG methylation post-enrichment for global scale non-CpG detection. (**C**) Single-molecule real-time sequencing: The recent development of single molecule real-time (SMRT) sequencing detects non-CpG methylation at single-base resolution without any enrichment techniques. Coupling SMRT with an algorithm that is specific for non-CpG methylation helps in detecting the CA/CT/CC methylation from CG methylation.

The detection of non-CpG methylation is tremendously improved due to the recently developed enzyme-based enrichment methods and sequencing strategies. Enzyme-based enrichment is sequence-specific but an inexpensive method of selectively separating methylated DNA fragments from unmethylated DNA fragments (Figure 1). Methylation-sensitive restriction endonuclease (MSRE) can recognize methylated DNA and can be categorized based on its sensitivity toward nucleotide base sequence. MSREs such as Acc II, Cpo I, HhaI, and Nru I were found to be sensitive to methylated CGs. While other enzymes such as Psp6 I, Ajn I, ApeKI, BbvI, EcoP15I, Fnu 4HI, MspI, MwoI, and TseI were found to restrict specifically at the non-CpG methylated sites [20]. Several MSREs can aid in the detection of non-CpG methylated sites however, MspI was found to be more specific in detecting non-CpG methylation with a wide range of CpA, CpC, and CpT methylation. The MSRE- PCR displays a moderate to high sensitivity and specificity [21]. Real-time PCR or high throughput sequencing are also used to quantitatively access non-CpG methylation levels. An improved MSRE method is the LUMA assay (Luminometric-based assay for global DNA methylation) which utilizes luminescence to detect non-CpG methylation. Here, the genomic DNA is digested with methylation-sensitive and -insensitive restriction enzymes followed by the luminometric polymerase extension, and the base modification is detected. MSREs Psp6I and Ajn I were used with LUMA for the detection of non-CpG methylation in the CCWGG context. A major limitation of LUMA is that it can be used to identify the global non-CpG methylation but not locus-specific sites [22,23,24]. A milestone in the timeline of methylation analysis is the bisulfite (BS) conversion method. During the bisulfite treatment, methylated cytosines remain intact while the unmethylated cytosine converts into uracil by deamination. Therefore, methylation-specific primers accompanied with bisulfite PCR would detect the non-CpG methylation in a targeted fragment. The sensitive primers and partial bisulfite conversion are two major limitations of the BS method [25]. Meissner et al. (2008) used non-conversion filtering with the BS-seq [26]. Nevertheless, the partial conversion of the BS reaction can be normalized with the experimental control of unmethylated lambda DNA, and also by other non-BS-dependent methods [27,28,29]. An advanced version of BS-PCR is hairpin-bisulfite PCR which detects the cytosine methylation patterns on complementary DNA strands using a hairpin linker. These hairpin linker sequences are 25–26 nt in length and are added to the restriction digested genomic DNA [30]. This technique overcomes the primer bias due to specificity in the PCR synthesis based on the linker sequences. On the other hand, the enrichment methods were found to actively help in the detection of non-CpG methylation.

### 2.3. Coupling Enzyme-Based Enrichment Strategies with High Throughput Sequencing

Genome-wide screening of non-CpG methylation distribution is a major shortcoming of MSRE. Therefore, merging the enzyme-based enrichment strategies with the high throughput sequencing techniques will result in a whole-genome detection of non-CpG methylation. Several enrichment techniques would identify the genome-wide distribution of non-CpG methylation (Figure 1). Bisulfite sequencing calculates the methylated cytosine ratio by comparing the methylation levels of methylated cytosines and unmethylated cytosines. [23]. Although BS-seq detects both the CpG and non-CpG methylated sites, the specificity and sensitivity are much higher in CpG detection when compared to non-CpG detection. Hair-pin bisulfite sequencing reliably produces higher specificity than the BS-seq with single-base resolution [13,14,31]. The specificity of non-CpG methylation detection by WGBS is highly dependent on the use of known controls to filter the background noise. Hence, WGBS is one of the most reliable techniques in the detection of non-CpG methylation Hence, there is a need of reforming the existing bioinformatic pipelines which will reveal detailed information about both genome-wide CpG and non-CpG methylation patterns at single-base resolution. The advancement of BS-seq is reduced representation bisulfite sequencing (RRBS) which measures genome-wide methylation patterns. RRBS exploits the restriction digestion with MspI followed by the bisulfite conversion and high-throughput sequencing. In 2011, Ziller et al. revealed the use of RRBS in non-CpG methylation detection (CpA, CpC, and CpT nucleotides) with higher sensitivity. The study showed that WGBS picked up 250,000 non-CpG loci while RRBS detected 213,000 non-CpG regions with an overlap of only 52,000 loci [3]. Later, several studies incorporated the RRBS method for the detection of non-CpG methylation over CpG methylation [32,33,34]. The MspI-based RRBS method largely enriches CpG methylation compared to non-CpG methylation. WGBS can be a preferable method over RRBS when it is employed with robust controls in the detection of non-CpG methylation. Olova et al. (2018) illustrated that WGBS would reduce the false positive methylation calls and the conversion artifacts in the detection of non-CpG methylation by using the BS controls [29] (Figure 1). Integrating enzyme-based enrichment methods with high-throughput technologies provides immense importance in the identification of non-CpG methylation.

### 2.4. Antibody-Based Enrichment Methods

The antibody which specifically captures non-CpG methylation is yet to be developed. The antibody against methylated cytosines captures both CpG and non-CpG methylation. Methylated DNA enrichment can be performed by two approaches: methylated DNA immunoprecipitation (MeDIP) and the methyl-CpG binding domain (MBD) protein capture method. MeDIP exploits the use of monoclonal antibodies that are specific to methylcytosine (5MeC14), while the MBD approach captures the double-stranded methylated DNA fragments with the help of MBD beads. The efficacy of both techniques majorly relies on antibodies that are specific to methylcytosine capture. Previous reports suggest 5-methylcytidine effectively binds in the low CpG methylated areas whereas the MBD binds to the high CpG methylated areas [33,35,36,37,38] (Figure 1). The antibody for 5-methylcytidine has binding to non-CpG methylated regions with low sensitivity. However, the antibody binding to the methylated sequence of DNA cannot discriminate between CpG and non-CpG sequences. This is due to its binding sensitivity towards symmetric CG repeats and the asymmetric non-CpG methylated cytosines. Hence, post-enrichment and sequencing, the methylated DNA was filtered using algorithms that can distinguish CpG and non-CpG contents.

### 2.5. The New Era of Non-CpG Methylation Detection

The development of single-molecule real-time (SMRT) sequencing has enabled a hyper-resolution analysis of rare and scattered nucleotide variations. The application of SMRT can be extended to methylation analysis with its advantage to read CHG amidst CpG sequences. Raw reads that were obtained from the long-read sequencing were base and variant called using Megalodon tools. The reads from several samples were pooled to plot the enriched heatmaps. Goldsmith et al., (2021) revealed a novel tool that would detect the pattern of methylation along the 16 kb reads with the coverage of >1000× spanning the entire mitochondrial chromosome [39]. Thus, it would help to eliminate PCR amplification bias followed by bisulfite conversion. There are several bioinformatic pipelines that are available to deduce the base and sequence variants for CpG methylation (Figure 1). Due to the lack of information on non-CpG methylation at single-base resolution, the exact functional impact is underestimated. Developing these long-read sequencing with deeper coverage exclusively for mCpH would shed light on the precise mechanism of non-CpG methylation.

## 3. Computational Approaches for Non-CpG Methylation

**Figure 2:**
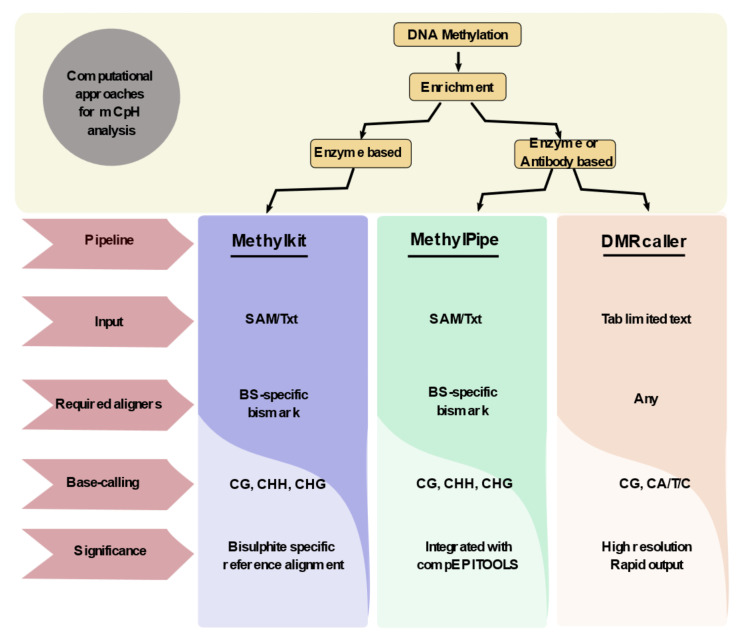
Computational approaches that are involved in non-CpG methylation analysis. DNA methylation analysis uses a computational pipeline to distinguish between non-CpG methylation and CpG methylation. There are three major bioconductor packages such as Methyl Kit, Methyl Pipe, and DMR caller that could identify non-CpG methylation with different types of factors such as input sequence (SAM/txt/tab-limited), different aligners (BS-Specific/general), and various base-calling strategies. All of these factors help in distinguishing non-CpG methylation with multiple advantages.

Computational biology plays a major role in the detection of non-CpG methylation due to the availability of a large set of human genomic data. Although enrichment techniques are equally important in differentiating non-CG from CG methylation, it does not reveal the sequence-specific information. Hence, integrating the computational approaches with the available techniques would aid in detecting non-CG methylation at a single-base resolution. The enrichment strategies can be classified into two groups based on the specificity of non-CpG detection: high-resolution and low-resolution enrichment techniques. High-resolution techniques include WGBS, RRBS, SMRT, Padlock, etc., whereas the MBD and MeDIP were grouped under low-resolution techniques. By exploiting the advantages of computational approaches, it would aid us to detect the undeciphered non-CpG methylation even from the low-resolution techniques. The development of computational tools and aligners aid in the detection of non-CpG methylation. Bismark discovery by Krueger et al., (2010) is one of the major milestones in identifying non-CpG methylation from the BS-seq data. Bismark employs the methylation calls in sequence context to classify the CpG, CHH, and CHG reads. The tools align the reads to the bisulfite reference genome to deduce the CpG and non-CpG methylated DNA regions. The integration of Seqmonk with bismark enables the visualization of BS-specific peak sets [40]. More than 38 packages are available in Bioconductor for the detection of DNA methylation, amidst only a few packages that can detect the non-CpG methylation in CHH and CHG context from the human genome. Akalin et al., (2012) developed a methyl Kit package that could provide information on CpG, CHG, and CHH from SAM files [41]. Later, Kishore et al., (2015) developed an integrative analysis tool methylPipe that could efficiently identify methylated cytosines and the oxidized form 5-hmC in both CpG and non-CpG contexts. Also, this pipeline provides basic knowledge on the absolute methylation (mC/bp) and relative methylation (mC/C) values [42]. All these packages such as methylPipe and methylKit are involved in making tiling bins by breaking the genome and followed by integrating the cytosine methylation levels in all bins. Most of these methods rely on the integrity and versatility of the enrichment techniques and are quite compromising. In 2018, Catoni et al. identified DMRcaller as an R package that could compute differentially methylated regions in both CpG and non-CpG contexts. The major facilitation of this package is it could consider the peaks that were called from the bisulfite sequencing as an input. It showed higher accuracy and reproducibility than the available packages and also generates DMRs with a faster computing time (within a few hours) using WGBS data [43]. Altogether, DMRcaller was found to be an efficient package in detecting and visualizing non-CpG methylation. In 2020, Teng et al. founded the first web tool MethGET which helps in correlating gene expression with CpG and non-CpG (CHG and CHH) methylation based on WGBS data [44]. Altogether, computational approaches for detecting non-CpG methylation are limited and it is important of developing new algorithms and web tools to detect and distinguish non-CpG methylation from CpG methylation in the existing datasets (Figure 2).

## 4. Current Challenges and Future Perspectives

Non-CpG methylation has not been extensively studied and its functional role is still unclear. The detection of non-CpG methylation among the widespread CpG methylation has been a tremendous task. Although several experimental and computational approaches can detect non-CpG methylation, eliminating the background CpG methylation remains a fundamental challenge. Recently developed enzyme-based enrichment methods and antibody-based enrichment methods are most often used for detecting non-CpG methylation. Coupling the enzyme-based enrichment method along with the antibody-based pull-down significantly enriches the non-CpG content. Alternatively, enrichment of non-CpG methylation with HpaII and immuno-precipitation can specifically enrich non-CpG without any CpG background. On the other hand, developing an algorithm to retrospectively filter non-CpG methylation of available whole-genome bisulfite sequence data may facilitate the characterization of tissue or tumor-specific non-CpG profiles. Similarly, MeDIP and MBD sequencing can be used to distinctively and simultaneously identify both non-CpG and CpG methylation. Revolutionized methodologies and reformed computational approaches can revamp the non-CpG methylation research and portray its unknown significance in human diseases.

## Data Availability

Not applicable.

## Abbreviations

5-mC	5-methylcytosine
DNMT	DNA methyltransferases
PCR	Polymerase chain reaction
MBD	Methyl CpG binding domain captures protein
MeDIP	Methylated DNA immunoprecipitation
LUMA	Luminometric methylation assay
BS-seq	Bisulfite sequencing
RE	Restriction enzyme
WGBS	Whole-genome bisulfite sequencing
RRBS	Reduced representative bisulfite sequencing
SMRT	Single-molecule real-time sequencing
